# Plasma-polymerized pericyte patches improve healing of murine wounds through increased angiogenesis and reduced inflammation

**DOI:** 10.1093/rb/rbab024

**Published:** 2021-06-30

**Authors:** Hannah M Thomas, Parinaz Ahangar, Robert Fitridge, Giles T S Kirby, Stuart J Mills, Allison J Cowin

**Affiliations:** 1 Future Industries Institute, University of South Australia, Mawson Lakes SA 5095, Australia; 2 Cell Therapy Manufacturing Cooperative Research Centre, Adelaide SA 5000, Australia; 3 Faculty of Health and Medical Sciences, University of Adelaide, Adelaide SA 5005, Australia

**Keywords:** pericytes, cell therapy, wound healing, plasma polymerization, advanced surfaces

## Abstract

Pericytes have the potential to be developed as a cell therapy for the treatment of wounds; however, the efficacy of any cell therapy relies on the successful delivery of intact and functioning cells. Here, the effect of delivering pericytes on wound repair was assessed alongside the development of a surface-functionalized pericyte patch. Plasma polymerization (PP) was used to functionalize the surface of silicone patches with heptylamine (HA) or acrylic acid (AA) monomers. Human pericytes were subsequently delivered to murine excisional wounds by intradermal injection or using the pericyte-laden patches and the comparative effects on wound healing, inflammation and revascularization determined. The AA surface provided the superior transfer of the cells to de-epidermized dermis. Excisional murine wounds treated either with pericytes injected directly into the wound or with the pericyte-laden AA patches showed improved healing with decreased neutrophil infiltration and reduced numbers of macrophages in the wounds. Pericyte delivery also enhanced angiogenesis through a mechanism independent of VEGF signalling. Pericytes, when delivered to wounds, improved healing responses by dampening inflammation and promoting angiogenesis. Delivery of pericytes using PP-AA-functionalized patches was equally as effective as direct injection of pericytes into wounds. Pericyte-functionalized dressings may therefore be a clinically relevant approach for the treatment of wounds.

## Introduction

The identification of cells that possess attributes that will enhance wound healing has been a long-term goal for the development of cell therapies. Pericytes are one such cell that has been considered for the treatment of muscle, bone and heart injuries [1–3], but pericyte effectiveness as a cell therapy for the treatment of wounds has yet to be determined. Pericytes are peri-vascular mesenchymal stem cell (MSC)-like cells involved in the regulation of both vascularization and inflammation, which have been shown to be important contributors to a number of processes important for successful wound healing [4, 5]. Pericyte presence on or near vascular structures regulates the infiltration of leukocytes from the blood stream to the site of injury [6, 7] and consequently is important in mediating the balance between an appropriate inflammatory response and the tissue damage which can result if this response is too severe. Pericytes are also important regulators of angiogenic activity and impact the capacity of a tissue to successfully re-vascularize after injury [8].

In diabetic patients, many tissues exhibit a decrease in pericyte populations, and this reduction has been implicated in the progression of some diabetic pathologies including diabetic foot ulcers [9]. Given that pericyte depletion is observed in the skeletal muscle of diabetic feet [10], it is possible that pericytes may also play a role in contributing to the delayed cutaneous healing experienced by diabetic patients. As a result, we have hypothesized that pericytes represent a population of cells which are beneficial for cutaneous healing and have the potential for development as candidates for the treatment of wounds.

The identification and characterization of beneficial cell types is not the only step in developing a successful cell therapy. A cell-based product must be efficiently delivered to the site of intended action, and difficulties in this process have the potential to impact the viability or efficacy of the intended therapy [11]. The simplest and most direct method of cell delivery is by injection immediately at the site of injury. This approach has been utilized in the preclinical and clinical testing of many cell therapies [12–14] and confers the benefit of allowing direct application of the cells to the intended location without relying on the cell itself to identify and reach the site of injury. There are downfalls to this delivery method however, as injection of cells has been shown to decrease cell viability. In fact, pushing cells through a syringe or needle has in some cases resulted in cell viabilities of only 1–32% [15]. The manual stress of syringe delivery can cause irreversible and sometimes fatal damage to the cell membrane [16]: not only does this negate the potential benefit of the cell therapy but also the introduction of a large population of apoptotic or necrotic cells may serve to elicit an immune response which could be detrimental to the healing process. Additionally, biomechanical stress can induce the secretion of pro-inflammatory factors in some cell types, such as MSCs [17]. Hydrogels [18], scaffolds [19], collagen plugs [20] and cell sheets [21], have previously been explored as alternatives to injection. Another possible method of cell delivery to the skin is by way of culturing the cells on a surface which will support their survival and can be applied, much like a dressing. Unlike injections, which are often applied around the margins of the wound, treatment with a dressing can have the additional benefit of supplying cells to the whole of the wounds without having to rely on cell migration into the wound site. Application of a cell-coated surface to an open wound can facilitate transfer of the cells from the surface into the wound bed, provided that the surface is tuned such that the cells will find the skin preferable and move from the surface into the wound. One way of functionalizing such surfaces is by plasma polymerization (PP) [22]. This is a process which distributes a chosen monomer in an even coating onto a surface, and can be used to coat medically approved silicone surfaces in a short time at a low cost [23]. The effects that surface chemistries can have on pericyte adhesion and proliferation have been investigated using plasma treatment and ion radiation [24]. There are, however, no studies to date where plasma polymerization has been used to deposit monomers onto a surface to improve adherence of pericytes and to then deliver the pericytes onto the surface of the skin or into a wound. Keratinocytes [25], MSCs [26], corneal epithelial cells [27] and multipotent adult progenitor cells (MAPCs) [28] have all been successfully delivered on PP-functionalized silicone to planar biological surfaces in pre-clinical murine trials. In this study, we cultured human pericytes on PP-functionalized surfaces and assessed the benefits of this method for pericyte delivery to wounds in comparison with direct injection.

## Materials and methods

### Preparation of plasma-polymerized patches

In order to develop a more clinically acceptable method of pericyte administration, plasma polymerization (PP) was used to create functionalized surfaces on silicone dressings, on which pericytes could be grown and delivered. Heptylamine (HA) and acrylic acid (AA) monomers were used to create amine-based and acid-based polymers on the surfaces. Silicone backing (Polymer Systems Technology, High Wycombe, UK) was cut into 15 mm × 15 mm squares and placed in a petri dish. The dish was placed in the chamber of a parallel plate reactor. Plasma polymerization was performed as previously described [28]. Briefly, following degassing with three freeze–thaw cycles, polymerization was carried out using precursors acrylic acid (AA) or heptylamine (HA) (Sigma Aldrich, Castle Hill, NSW, Australia). A rotary pump was used to evacuate the reaction chamber to a base pressure of less than 1 × 10^−4^mbar. With an initial reaction pressure of 2 × 1 0 ^−2^ mbar, deposition was carried out for 20 mins at a power of 5 W. Samples were stored in sealed petri dishes for analysis and subsequent use.

### X-ray photoelectron spectroscopy

To determine the predominate residues present on the surface of the plasma-functionalized patches, a SPECS SAGE X-ray photoelectron spectroscopy (XPS) system was used to obtain X-ray photo-electron spectra with an Mg Kα radiation source operating at 10 kV and 20 mA. A survey spectrum between 0 and 1000 eV was taken to assess all elements present on the sample surface. High-resolution spectra were subsequently taken for selected peaks. Processing and deconvolution of the spectra were carried out using CasaXPS software (Neal Fairley, UK).

### Cell culture

Human placental pericytes (hPC-PL) (Promocell, Heidelberg, Germany) were cultured in pericyte media (Promocell, Heidelberg, Germany) supplemented with 1% penicillin streptomycin (Sigma Aldrich, Castle Hill, NSW, Australia) and maintained at 37°C with 5% CO_2._

### MTT transfer assay

hPC-PLs at the density of 1 × 10^5^ were seeded in stainless steel rings on patches in a 6-well plate (Corning, New York, USA) and allowed to grow for 24 hours. The stainless steel rings and media were removed and 2 ml of 0.5 mg/mL MTT reagent (3-(4,5-dimethylthiazol-2-yl)-2,5-diphenyltetrazolium bromide) (Life Technologies Australia, Scoresby, VIC, Australia) in PBS was added to each well. The cells were incubated for 4 h at 37°C to assess the metabolic activity of cells on each patch. To assess the capacity of each patch for cell transfer to skin *in vitro*, cells were seeded on patches as described above and grown for 24 h. De-epidermized human acellular dermis (DED) was obtained from the European Skin Bank and prepared with three overnight PBS washes followed by one-hour incubation at 37°C in 1 M sodium chloride solution to remove the epidermis and preserve membrane proteins. The DED was rehydrated in Dulbecco’s modified eagle media (DMEM) (Gibco, Thermo Fisher Scientific, Scoresby, VIC, Australia), cut into 15 mm  × 15 mm squares and placed in a 6-well plate. Pericyte-laden patches were placed carefully face down on the DED and weighted with a stainless steel ring to ensure continued contact. Wells were filled with 2 ml media and left for 24 h after which the rings were removed, the patches carefully lifted from the DED and placed in clean wells. 2 ml of 0.5 mg/mL MTT reagent was added to each well and incubated for 4 h at 37°C to assess the transferred patches and DED for the presence of metabolically active pericytes.

### Pericyte delivery to murine excisional wounds

These studies were conducted with approval from the Women’s and Children’s Health Network (WCHN) Animal Ethics Committee (AEC) and carried out at the WCHN animal facility in keeping with the Australian Code of Practice for the Care and Use of Animals for Scientific Purposes (AEC: 1090.05.2021). Twelve-week-old Balb/C female mice from the Animal Resources Centre (ARC) (Canning Vale, WA, Australia) were acclimatized for 1 week prior to excisional wounds being created as described previously [29]. Briefly, following anaesthesia, the dorsa of mice were shaved, treated with Veet and swabbed with ethanol to remove all hair. Two 6-mm full-thickness wounds were created using a 6 mm Acu-Punch Biopsy Punch (EBOS, Kingsgrove, NSW, Australia) 5 mm either side of the midline and 10 mm from the base of the skull. Pericytes (hPC-PL) were applied to the excisional wounds immediately after wounding by way of injection or patch application. Pericytes for injection were suspended in PBS (either 2 × 10^4^ or 8 × 10^4^ cells in 100 µl) and injected intradermally at four points (25 µl/injection) around the periphery of the wound immediately after surgery using a 22 G needle. Control wounds were injected with an equivalent volume of PBS alone. Injected wounds were covered with Tegaderm (3 M, North Ryde, NSW, Australia) for 3 days, after which the dressing was removed. Pericytes for patch application were pipetted into stainless steel rings on patches (2 × 10^4^ or 8 × 10^4^ cells/patch) 24 h prior to surgery. The patches were applied cell-side down to the wound immediately after surgery and affixed to the dorsa of the mice with Tegaderm dressing. The patch and dressing remained for 3 days, after which they were removed. Any mice who lifted or removed their patches/dressings during the first 3 days were anaesthetized and the patches restored. Wounds were left to heal for 7 days and then collected for analysis (*n* = 6 mice, 12 wounds for each treatment group).

### Wound measurement

On the day of surgery and wound collection, a ruler was aligned with each wound and digital images were collected to allow the measurement of macroscopic wound area. Images were imported into Image ProPlus (Media Cybernetics Inc., Bethesda, MD, USA) software, and measurement tools were calibrated for each image using the ruler in the frame. Wound area at the time of collection (Day 7) was measured, normalized to the original wound area measured and mean macroscopic wound area calculated for each group. Histological wound analysis was carried out manually using cellSens software (Olympus, Tokyo, Japan). Bright-field images of haematoxylin and eosin-stained sections were assessed for dermal wound width and the extent of re-epithelialization. Wound width was defined as the measurement between the left and right outer edges of the dermal wound area. Re-epithelialization was calculated as the percentage of the total epithelial length covered by the two migrating tongues of the neoepidermis. Mean microscopic wound width and re-epithelialization measurements were calculated for each group.

### Immunofluorescence

Sections were deparaffinized in two 15-min changes of xylene and brought to water through two 2-min changes in absolute EtOH, 2 min in 70% EtOH, 2 min in 30% EtOH and 4 min in tap water. Sections were washed in PBS and transferred to a decloaker (Biocare Medical, Pacheco, CA, USA) for antigen retrieval (heating in citric acid buffer at 90°C for 10 min) followed by a 3 min immersion in 0.25 g/L trypsin (Sigma Aldrich, Castle Hill, NSW, Australia) in PBS at 37°C. Sections were washed in PBS and then blocked for 1 h in 3% serum (Sigma Aldrich, Castle Hill, NSW, Australia) in PBS, with the source of the serum corresponding to the species in which the intended secondary antibody was raised. The blocking solution was then replaced with the primary antibody: NIMP Santa Cruz sc-59338 Rat 1:200, F4/80 Bio-Rad MCA4975 Rat 1:200, VEGF Abcam ab46154 Rabbit 1:200, CD31 Abcam ab28364 Rabbit 1:200 or HNA Abcam ab190710 Mouse 1:200 diluted in 3% serum, and sections were left to incubate overnight at 4°C. Sections were subsequently washed three times for 5 min in PBS and then incubated for 1 h at room temperature in secondary antibody diluted in PBS. Sections were stained for 5 min in 1:5000 4ʹ,6-diamidino-2-phenylindole (DAPI) (Sigma Aldrich, Castle Hill, NSW, Australia) in PBS, washed three times for 5 min in PBS, dried and mounted in PermaFluor mounting media (Thermo Fisher Scientific, Scoresby, VIC, Australia). Stitched images of the entire wound area were acquired for each sample and analysed using cellSens software. For intensity measurements, mean grey intensity was measured across the wound bed and the auto-fluorescent measurement taken from a matched no primary control section was subtracted. For cell counts, the area of interest was manually defined and measured. The number of positive cells within the area of interest was manually counted and then normalized to the area measurement for each sample. Mean measurements were calculated for each wound group. Human skin samples were used as positive controls for HNA staining. Human skin samples were collected from patients at the Queen Elizabeth Hospital with approval from the Health Service Human Research Ethics Committee and the Central Northern Adelaide Health Service Ethics of Human Research Committee (EC00190). All studies were carried out in accordance with the Declaration of Helsinki (1964).

### Statistical analysis

For macroscopic comparisons between pericyte treatment and control groups, statistical significance was calculated using a two-way ANOVA with multiple comparisons. For histological and immunohistochemical comparisons between these wounds, statistical significance was calculated using a Student’s *t*-test. Annotation of significance above any treatment group indicates statistical significance between that treatment and the relevant control group. For all analyses, any *P* values of <0.05 were considered significant. Graphical representation of significance: *P < 0.05, **P < 0.01, ***P < 0.001, ****P < 0.0001. All data are displayed as mean ± SEM.

## Results

### Pericytes injected into acute wounds improve healing

**Figure 1.**
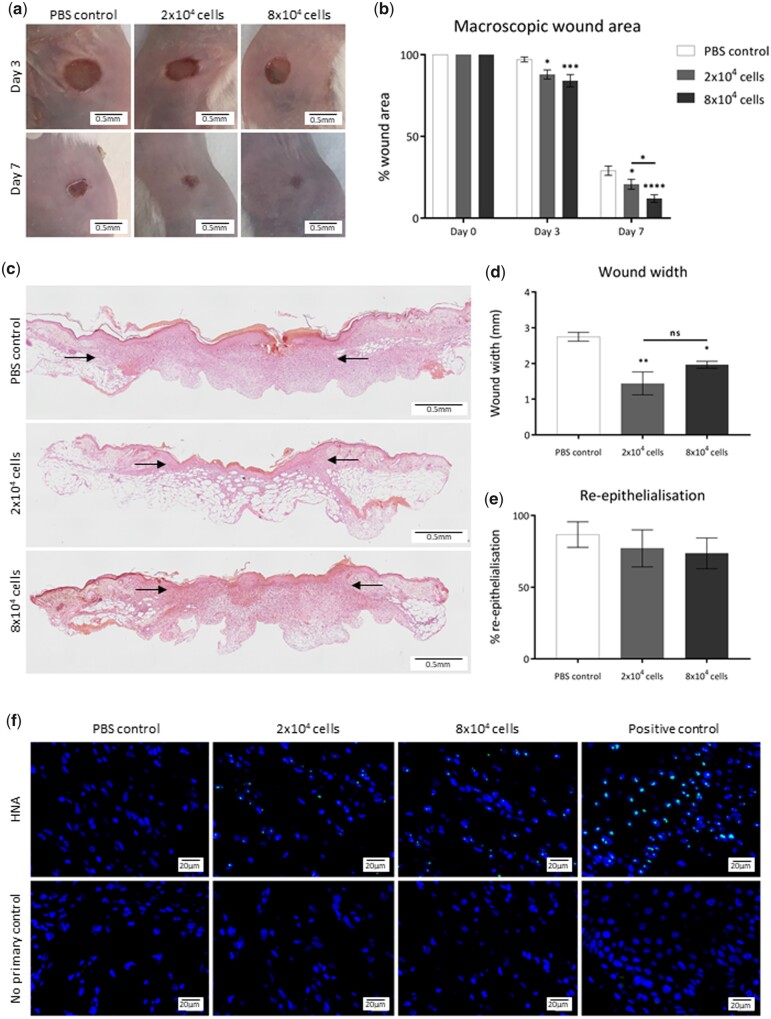
Injection of pericytes improves healing of acute murine wounds. (**a**) Representative images of wounds injected with 2 × 10^4^ pericytes, 8 × 10^4^ pericytes or PBS alone, 3 and 7 days after wounding. Black scale bars represent 5 mm. (**b**) Quantification of macroscopic wound area. Statistical significance calculated by two-way ANOVA, where *P < 0.05, ***P < 0.001, ****P < 0.0001. (**c**) Haematoxylin and eosin-stained sections representative of wounds injected with 2 × 10^4^ pericytes, 8 × 10^4^ pericytes or PBS alone, 7 days after wounding. Composite images captured at 10× objective, black scale bars represent 0.5 mm. (**d**) Quantification of histological wound width. (**e**) Quantification of re-epithelialization calculated as a percentage of total epithelial length. Statistical significance calculated by one-way ANOVA, where *P < 0.05, **P < 0.01. (**f**) Images representative of HNA-positive (green) cells counterstained with DAPI (blue) in PBS control, 2 × 10^4^ and 8 × 10^4^ pericyte injected wounds, 7 days after wounding. Positive control: acute human wound sample. Images captured at 40× objective, white scale bars represent 20 μm. *n* = 6 for all groups, all data are represented as mean ± SEM. Annotation of significance above any treatment group indicates statistical significance between that treatment and the PBS control group at that time point

In order to be able to compare the efficacy of pericytes delivered by PP-functionalized patches to those directly injected into wounds, we first performed an assessment of direct injection into murine excisional wounds. Commercially available human pericytes of placental origin (hPC-PL) were delivered to excisional wounds in Balb/C mice by intradermal injection. Wounds received either 2 × 10^4^ cells in PBS (low dose), 8 × 10^4^ cells in PBS (high dose) or a control injection of PBS only immediately after wounding, at four sites around the periphery of the wound. Wounds were covered with a Tegaderm dressing for 3 days, after which the dressing was removed. Macroscopic measurements of wound area were calculated from images taken at Days 3 and 7 and normalized to original wound size for each sample (Fig. 1a and b). Wounds injected with both low and high cell doses displayed a significant acceleration in wound closure compared to the PBS-injected control wounds: both treatment groups displayed significantly smaller macroscopic wound areas than the PBS control at Days 3 and 7 of healing. At Day 7, the average wound area of the high-dose treatment group was significantly reduced (11.96% of initial wound area) compared to that of the low-dose-treated wounds (20.64%) and PBS control (29.02%). Histological assessment of dermal wound width in haematoxylin and eosin-stained paraffin-embedded Day 7 wounds (Fig. 1c) revealed that wound width was significantly decreased in both the low- and high-dose treatment groups when compared to the PBS control group (Fig. 1d). While wound area and width measurements suggested improved healing, there was no specific effect on % re-epithelialization at Day 7 post-wounding (Fig. 1e). Staining of paraffin-embedded Day 7 wounds with an anti-human nucleoli antibody (HNA) allowed visualization of the human pericytes within the murine wounds. Both low- and high-dose-injected wounds displayed the presence of HNA-positive cells in the wound bed, confirming pericytes delivered at Day 0 remained in the wound site until at least 7 days after injection (Fig. 1f).

### Plasma-polymerized silicone patches functionalized with acrylic acid effectively transfer pericytes to human skin *in vitro*

X-ray photoelectron spectroscopy (XPS) was used to characterize and verify the plasma polymer surface coatings (Table 1). Heptylamine (HA) plasma polymer is most easily confirmed by the presence of nitrogen as shown by an N1s peak at 399 eV and the suppression of silicon from the underlying silicone rubber. Previous characterization of the HA plasma polymer reported the N/C ratio of 0.05 at 5 W [28], and this analysis shows a similar ratio of 0.052. Similarly, acrylic acid (AA) plasma polymer has a reported O/C ratio of 0.36 at 5 W, and this analysis shows a similar ratio of 0.31. Furthermore, the characteristic carboxyl and alcohols are present upon deconvolution of the high-resolution C1s spectra (Supplementary Figure S1). The presence of silicon is typical due to the mobility of the silicone chains in the underlying rubber, the percentage of which can change slightly as the polymer ages. This characteristic was previously reported and had little-to-no effect on the utility of the polymer surface [28].

**Table 1.** Summary of XPS for surfaces generated using HA and AA precursors

Monomer	C1s	O1s	N1s	Si2p	O/C	N/C	COOH/R	C-OH
HA	85.63	6.659	4.48	3.231	0.078	0.052		
AA	65.577	20.341	0	14.082	0.31		12.99	10.45

**Figure 2.**
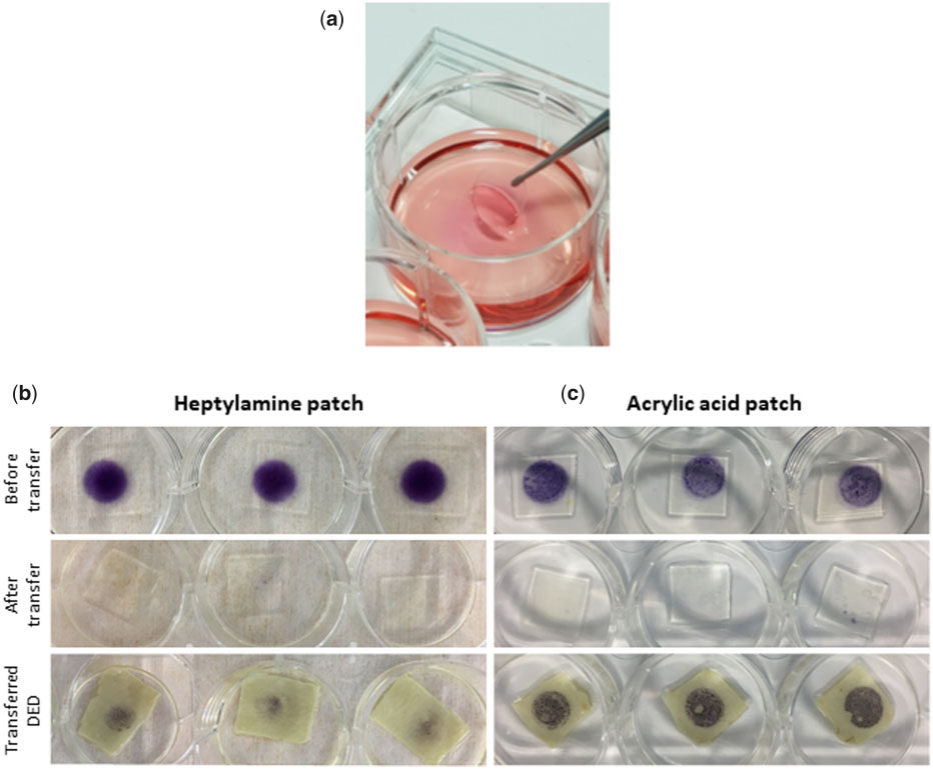
Plasma-polymerized silicone patches functionalized with acrylic acid effectively transfer pericytes to human skin *in vitro.* (**a**) Plasma-polymerized silicone patch. MTT transfer assay assessing hPC-PL delivery by (**b**) HA patch and (**c**) AA patch *in vitro*. Pictured are patches before transfer to DED, patches after transfer to DED and DED after patch application. MTT staining (purple) indicates the presence of metabolically active cells

Each surface was assessed for the capacity to support the attachment and survival of pericytes in culture using a metabolic MTT assay. Cell-coated PP-functionalized patches (Fig. 2a) were applied to de-epidermized acellular human dermis (DED) in an *in vitro* model of application to skin in order to investigate the ability of the pericytes to move from the functionalized surface of the PP-patch to the skin. Transfer assays indicated that the chemistry of both patches supported pericyte survival for 24 h (Fig. 2b and c). The application of HA PP-functionalized patches carrying pericytes to human DED did not result in efficient transfer of the cells from the PP-functionalized patch to the skin. As the cells also did not remain on the patch after transferring, it was assumed that cells not adhered to the DED were washed off into the media (Fig. 2b). In contrast, the AA surface performed considerably better, facilitating effective overnight transfer of the pericytes to DED (Fig. 2c). Consequently, the AA PP-functionalized patch was selected for non-invasive pericyte delivery to murine wounds *in vivo*.

### Plasma-polymerized acrylic acid patches effectively deliver pericytes into wounds, resulting in improved healing

**Figure 3.**
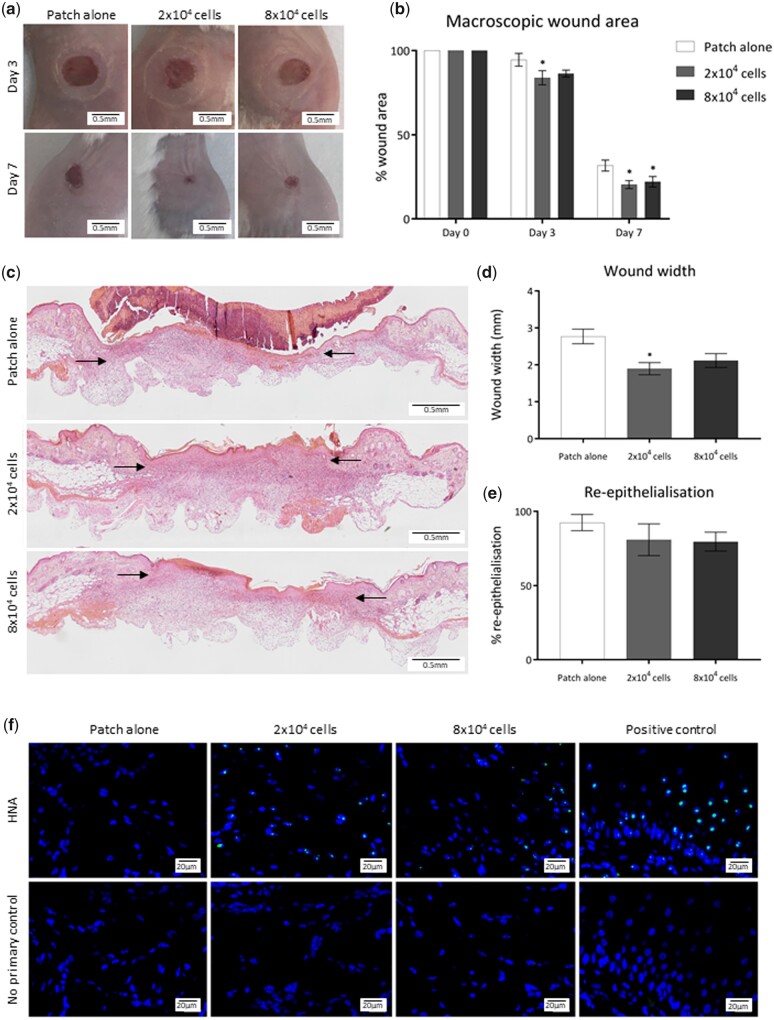
AA PP-functionalized patch delivery of pericytes improves healing of acute murine wounds. (**a**) Representative images of wounds treated with 2 × 10^4^ pericytes on PP-patch, 8 × 10^4^ pericytes PP-patch or PP-patch alone, 3 and 7 days after wounding. Black scale bars represent 5 mm. (**b**) Quantification of macroscopic wound area. Statistical significance calculated by two-way ANOVA, where *P < 0.05. (**c**) Haematoxylin and eosin-stained sections representative of wounds treated with pericyte PP-patch, 8 × 10^4^ pericyte PP-patch or PP-patch alone, 7 days after wounding. Composite images captured at 10× objective; black scale bars represent 0.5 mm. (**d**) Quantification of histological wound width. (**e**) Quantification of re-epithelialization calculated as a percentage of total epithelial length. Statistical significance calculated by one-way ANOVA, where *P < 0.05. (**f**) Images representative of HNA+ (green) cells counterstained with DAPI (blue) in 2 × 10^4^ pericytes on PP-patch, 8 × 10^4^ pericytes PP-patch or PP-patch-alone-treated wounds. Positive control: acute human wound sample. Images captured at 40 × objective; white scale bars represent 20 μm. *n* = 6 for all groups. Annotation of significance above any treatment group indicates statistical significance between that treatment and the patch-alone control group at that time point

hPC-PLs were delivered to excisional wounds of Balb/C mice using AA PP-functionalized patches. hPC-PLs were cultured on the AA PP-functionalized patches for 24 h prior to application to the wounds. Wounds received either 2 × 10^4^ cells (low dose), 8 × 10^4^ cells (high dose) or a control AA patch alone, immediately after surgery. The patches were secured with a Tegaderm dressing for 3 days, after which the patch and dressing were removed. Macroscopic assessment of wound size from images taken at Days 3 and 7 of healing (Fig. 3a and b) indicated that average wound area was significantly reduced in the low-dose pericyte PP-functionalized-patch-treated wounds at Days 3 (83.94%) and 7 (20.40%) (Fig. 3a and b and Table 2). High-dose pericyte PP-functionalized-patch-treated wounds were significantly smaller at Day 7 (22.08%) than patch-alone control wounds (31.72%). There was no significant difference between the wound areas of the high- and low-dose groups at Day 7 (Table 2). Histological measurements of haematoxylin and eosin-stained paraffin-embedded Day 7 sections (Fig. 3c and d and Table 2) indicated significantly decreased wound width in the low-dose PP-functionalized patch treatment group; however, the width of high-dose-patch-treated wounds was not significantly different to the average width of the PP-functionalized patch-alone control group (Fig. 3d and Table 2). Re-epithelialization was not impacted by pericyte delivery on the PP-functionalized patches, with wounds of all groups displaying comparable levels of epithelial restoration by Day 7 (Fig. 3e and Table 2). HNA staining of Day 7 wounds confirmed successful delivery of human pericytes to the wound bed, as HNA-positive cells were present in the wounds of both low- and high-pericyte PP-functionalized patch treatment groups (Fig. 3f and Table 2).

**Table 2.** Assessment of excisional wound healing in mice treated with pericytes injections and patch-coated pericytes

Healing assessment	Injection treatment		Patch treatment	
Macroscopic wound area (% initial wound)	**Groups**	**Day 7**	**Groups**	**Day 7**
	PBS control	29.02 ± 2.82	Patch control	31.72 ± 3.27
	2 × 10^4^ injection	20.64 ± 3.04	2 × 10^4^ patch	20.40 ± 2.39
	*P* values	0.0336	*P* values	0.0109
	8 × 10^4^ injection	11.96 ± 2.35	8 × 10^4^ patch	22.08 ± 3.11
	*P* value	< 0.0001	*P* value	0.0343
Microscopic wound width (mm)	PBS control	2.75 ± 0.12	Patch control	2.77 ± 0.20
	2 × 10^4^ injection	1.44 ± 0.32	2 × 10^4^ patch	1.90 ± 0.16
	*P* value	0.0013	*P* values	0.0111
	8 × 10^4^ injection	1.97 ± 0.10	8 × 10^4^ patch	2.12 ± 0.19
	*P* value	0.0431	*P* values	0.0581
Re-epithelialization (%)	PBS control	86.70 ± 8.94	Patch control	92.48 ± 5.46
	2 × 10^4^ injection	77.11 ± 12.93	2 × 10^4^ patch	80.91 ± 10.68
	*P* value	0.8129	*P* values	0.5627
	8 × 10^4^ injection	73.66 ± 10.70	8 × 10^4^ patch	79.65 ± 6.40
	*P* value	0.6848	*P* values	0.4958

Data are presented as mean ± SEM.

### Plasma-polymerized acrylic acid pericyte patches decrease inflammation in wounds

**Figure 4.**
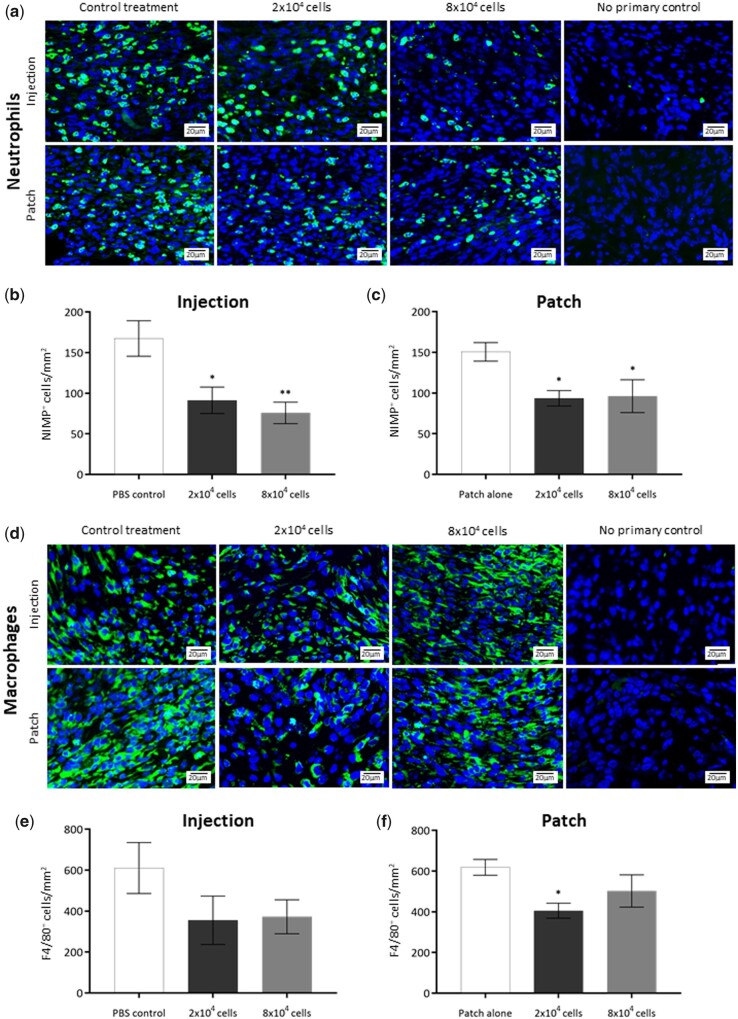
Effect of pericyte treatment on inflammatory cell markers in acute wounds. (**a**) Immunofluorescent detection of neutrophils by NIMP-R14 (green) counterstained with DAPI (blue) in Day 7 excisional wound sections treated with 2 × 10^4^ and 8 × 104 pericytes delivered either by injection or by PP-patch. (**b**) Quantification of NIMP-R14-positive neutrophils normalized to area in the wounds treated by pericyte injection. (**c**) Quantification of NIMP-R14-positive neutrophils normalized to area in the wounds treated by pericyte PP-patch. (**d**) Immunofluorescent detection of macrophages by F4/80 staining (green) counterstained with DAPI (blue) in Day 7 excisional wound sections treated with 2 × 10^4^ and 8 × 104 pericytes delivered either by injection or by PP-patch. (**e**) Quantification of F4/80-positive macrophages normalized to area in the wounds treated by pericyte injection. (**f**) Quantification of F4/80-positive macrophages normalized to area in the wounds treated by pericyte PP-patch. *n* = 6 for all groups, all data are represented as mean ± SEM. Statistical significance calculated by Student’s *t*-test, where *P < 0.05, **P < 0.01. Annotation of significance above the treatment group indicates statistical significance between that treatment and the PBS control group at that time point. Images captured at 40× objective; white scale bars represent 20 μm

Immunofluorescent staining was employed to assess the impact of pericyte delivery either by injection or by PP-functionalized patch on wound inflammation. NIMP-R14 staining was used to quantify neutrophil infiltration in pericyte-injected and PP-functionalized-patch-treated Day 7 wounds (Fig. 4a–c). Neutrophil numbers were counted and normalized to wound area for each sample. Pericyte-injected wounds displayed significantly decreased neutrophil numbers in the granulation tissue at Day 7 when compared to PBS-treated control wounds (Fig. 4a and b). Pericyte-patch-treated wounds also showed lower NIMP-R14-positive cells in the wounds (Fig. 4a and c) indicating that neutrophil infiltration was lower in pericyte PP-patch-treated wounds than patch-alone wounds at Day 7 (Fig. 4a and c). Macrophage infiltration was assessed using F4/80 staining (Fig. 4d), and cell counts were normalized to wound area (Fig. 4e and f). Pericyte-injected wounds displayed a trend towards decreased macrophage presence in the wound bed; however, this difference did not reach statistical significance (Fig. 4d and e). Pericyte PP-functionalized-patch-treated wounds displayed significantly decreased macrophage numbers in the wound beds treated with 2 × 10^4^ cells when compared with patch-alone control wounds (Fig. 4d and f).

### Plasma-polymerized acrylic acid pericyte patches increase vascularization of wounds

**Figure 5.**
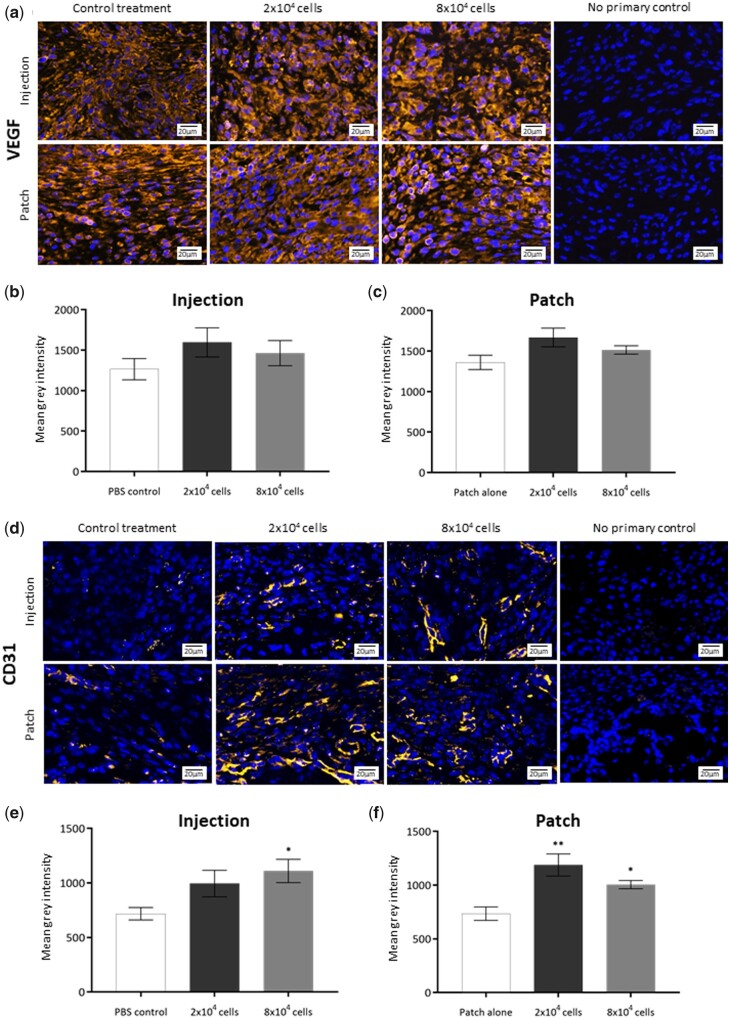
Effect of pericyte treatment on angiogenesis in acute wounds. (**a**) Immunofluorescent detection of CD31-positive endothelial cells (orange) counterstained with DAPI (blue) in Day 7 excisional wound sections treated with 2 × 10^4^ and 8 × 10^4^ pericytes delivered either by injection or by PP-patch. (**b**) Quantification of CD31-positive cells normalized to area in the wounds treated by pericyte injection. (**c**) Quantification of CD31-positive cells in the wounds treated by pericyte PP-patch. (**d**) Immunofluorescent detection of VEGF (orange) counterstained with DAPI (blue) in Day 7 excisional wound sections treated with 2 × 10^4^ and 8 × 10^4^ pericytes delivered either by injection or by PP-patch. (**e**) Quantification of VEGF treated by pericyte injection. (**f**) Quantification of VEGF in the wounds treated by pericyte PP-patch. *n* = 6 for all groups, all data are represented as mean ±SEM. Statistical significance calculated by Student’s *t*-test, where *P < 0.05, **P < 0.01. Annotation of significance above the treatment group indicates statistical significance between that treatment and the PBS control group at that time point. Images captured at 40× objective, white scale bars represent 20 μm

The impact of pericyte injection and pericyte PP-functionalized patch application on angiogenesis was assessed by immunohistochemical staining of VEGF and CD31 in the granulation tissue of the Day 7 wounds (Fig. 5). Staining was normalized to wound area and compared between low- and high**-**dose pericytes as well as control wounds. VEGF expression was compared between injected pericytes and PBS**-**injected wounds (Fig. 5a–c) and showed no significant difference observed between VEGF levels in treated or control wounds at Day 7 of healing (Fig. 5b). Similarly, pericyte delivery by PP-functionalized patch did not significantly impact VEGF expression within the wound bed (Fig. 5c). Injection of pericytes resulted in significantly increased CD31 expression in the wound bed when compared to wounds injected with PBS alone (Fig. 5d and e), which was statistically significant when the high dose (8 × 10^4^) of cells was injected (Fig. 5e). When the pericytes were delivered using the PP-functionalized patch, CD31 expression was significantly greater in pericyte PP-functionalized patch**-**treated wounds with both the low and high doses of cells when compared to control wounds (Fig. 5d and f).

## Discussion

The identification and development of cell therapies is an emerging and rapidly expanding focus for regenerative medicine. As most diseases and pathologies result from cellular death or dysfunction, using endogenous cells that have the capacity to contribute to tissue regeneration may be a way to address these pathologies. In recent years, pericytes have been identified as a potentially underutilized cell population with the capacity to regulate the behaviour of other cells and to regenerate cell populations of multiple lineages [30]. Pericytes have been applied to mouse models of bone and muscle injury [1, 2], ischaemic heart [3] and diabetic retinopathy [31], and show a promising capacity to contribute to the regeneration of these tissues, consistently accelerating tissue repair and causing increased revascularization. However, the identification of promising cells that promote tissue regeneration and repair is only one part of the solution: the method of cell delivery is a key aspect in the development of a successful cell therapy [32]. Systemic methods of delivery (e.g. intravenous, intraperitoneal or intraventricular injection) insert cells into the circulation, and consequently therapeutic efficacy relies on homing of those cells to the site of therapeutic need [33]. A preferred alternative is application of cells by injection directly to the injured tissue, as this confers immediate local action [34]. Apart from there being a significant level of efficacy, for clinical uptake of new technologies there is the added requirement that cell treatments are simple, requiring limited preparation and minimal training. The development of cell-based dressings is therefore an attractive approach for the delivery of cells into wounds.

PP-functionalized silicone surfaces have previously been explored to facilitate the delivery of multiple cell types. While keratinocytes and endothelial cells proliferate and migrate faster in response to a surface rich in acidic groups, dermal fibroblasts and MAPCs prefer an amine-based surface [22, 28]. Interestingly, while this same HA-functionalized PP-patch supported the survival of pericytes, it did not effectively transfer these cells to skin explants in culture. In contrast, when pericytes were cultured on acid-based AA patches, the cells survived and were efficiently transferred *in vitro*. Human pericytes were subsequently delivered to murine excisional wounds by both intradermal injection and use of the cell-laden AA PP-functionalized patches and the effect on wound healing determined.

Assessment of healing indicated that, independent of the method of delivery, pericytes caused an improvement in the rate of wound closure. Macroscopic wound area and histological wound width were decreased in wounds treated with low and high doses of pericytes. This may be due to differentiation of the pericytes into contractile myofibroblasts, as pericytes have been shown to contribute to this population of contractile cells [35, 36]. A previous study reported the application of pericytes in a collagen I plug caused a delay in dermal wound resolution [20], which contradicts the effects of pericyte delivery observed in this study. These differences in the effect of pericyte application may be due to the different methods of cell application and highlight the importance of the method of delivery: the insertion of collagen gels may have served to effectively stent the closure of the wounds, an effect which was not at play when the cells were administered by intradermal injection or delivered by PP-functionalized patches. Whilst there was little difference in the repair rates when the cells were treated either with pericyte injections or with the pericyte PP-functionalized patches, differences may occur when larger, or more complex wounds are investigated. In larger wounds, pericytes delivered via injection may be limited by the distance and they are able to migrate into the wound limiting their effectiveness whilst cell patches would deliver pericytes throughout the wounds. Pericyte-laden PP-functionalized patches could also, potentially, be frozen and stored for later use [37], which would greatly improve their usability in the clinic. While cell suspensions could also be used in the clinic, they generally contain significant volumes of DMSO, which can be toxic in wounds and would also require specially trained staff and equipment to prepare the cells for injection.

Re-epithelialization of wounds was not significantly affected by pericyte delivery by injection or PP-functionalized patches. *In vitro* studies have shown paracrine pericyte regulation of epithelial restoration by way of BMP-2 signalling, with increased pericyte presence in the dermal layer of organotypic cultures promoting accelerating formation of the epidermis [38]. The discrepancy between pericyte impact on epithelialization *in vitro* and *in vivo* may be due to an inability of delivered human pericytes to effectively signal and communicate with the endogenous murine cells of the wound environment.

Quantification of neutrophil and macrophage numbers in pericyte-treated wounds, delivered either by injection or by PP-functionalized patch, showed a reduction in inflammation. Endogenous pericytes regulate neutrophil extravazation in a number of tissues by remodelling the basement membrane of the blood vessel wall [6, 7], and delivery of exogenous pericytes to a murine model of the ischaemic heart decreases macrophage infiltration at the site of injury [3]. This capacity of pericytes to dampen inflammation has the potential to be of significant benefit during cutaneous healing, particularly in chronic wounds where excessive inflammation contributes to impaired healing [39].

Enhanced angiogenesis was also a feature of the wounds treated with pericytes delivered either by injection or on the PP-functionalized patches, with significantly greater expression of endothelial marker CD31 observed. This increase in CD31-positive endothelial cells is likely to be in response to either physical pericyte presence or paracrine signalling and suggests that increased pericyte presence alone can affect positive change in levels of wound angiogenesis. Other studies have delivered adipose-derived stem cells expressing perivascular markers αSMA, PDGFRβ, NG2 and Ang1 to rat wounds using fibrin gels and reported similar vascular benefits [40]. Enhanced neo-vascularization has also been observed in wounds treated with pericyte cell sheets and human umbilical cord perivascular cells (HUCPVCs) [41, 42]. Interestingly, VEGF expression was found to be unchanged in wounds treated with pericytes, indicating that pericyte delivery did not alter the expression of this pro-angiogenic signal within the wound bed. Pericytes have been reported to support the stabilization of vascular structures [43], and we have previously shown *in vitro* that this stabilization is in part physical and cannot be achieved through the delivery of pericyte-conditioned media alone [44], supporting the notion that delivery of an increased pericyte population may support the accumulation of stabilized vessels over the course of healing and enhance angiogenesis and revascularization through a mechanism independent of VEGF signalling.

This study has shown that pericytes have the potential to improve healing responses by dampening inflammation and promoting angiogenesis. The delivery of pericytes using AA PP-functionalized patches was equally as effective as direct injection of the cells into the wounds. The development of pericyte-based cell dressings may be a clinically relevant approach for the treatment of wounds which does not require injection.

## Supplementary Material

rbab024_Supplementary_Data
