# Supplementary material for: Plasma-polymerized pericyte patches improve healing of murine wounds through increased angiogenesis and reduced inflammation
Source: Regen Biomater. 2021 Jun 30;8(4):rbab024. doi: 10.1093/rb/rbab024 (PMC8242226; doi:10.1093/rb/rbab024)
Supplement: rbab024_Supplementary_Data [file rbab024_supplementary_data.docx]

**Supplementary Figure 1. Patch development and validation.** XPS analysis of heptylamine (HA) patch (a, b) confirming the deposition of an amine surface. XPS analysis of acrylic acid (AA) patch (c, d) confirming the deposition of an acidic surface.


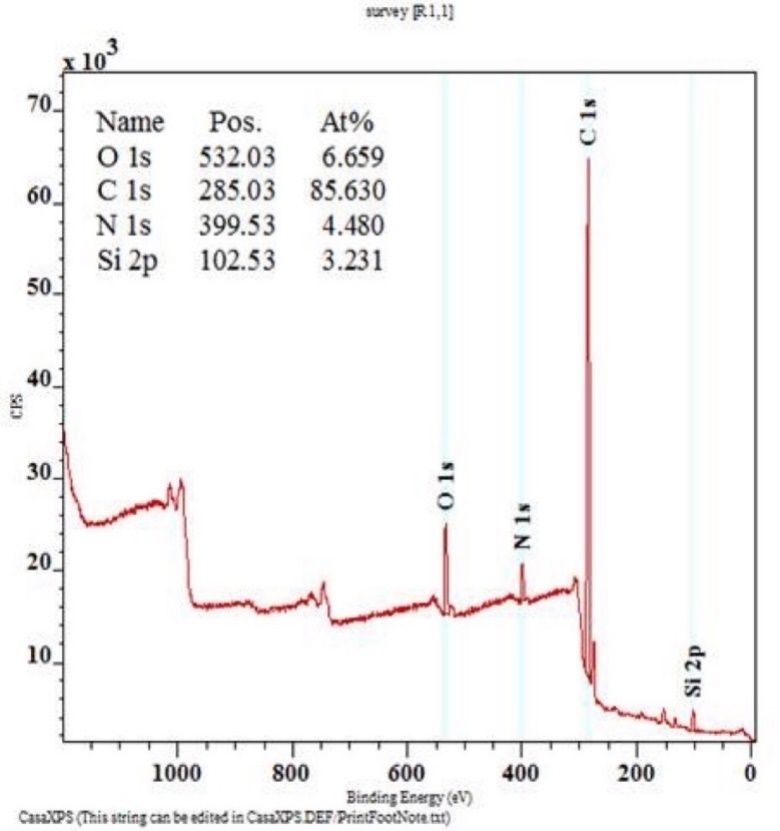

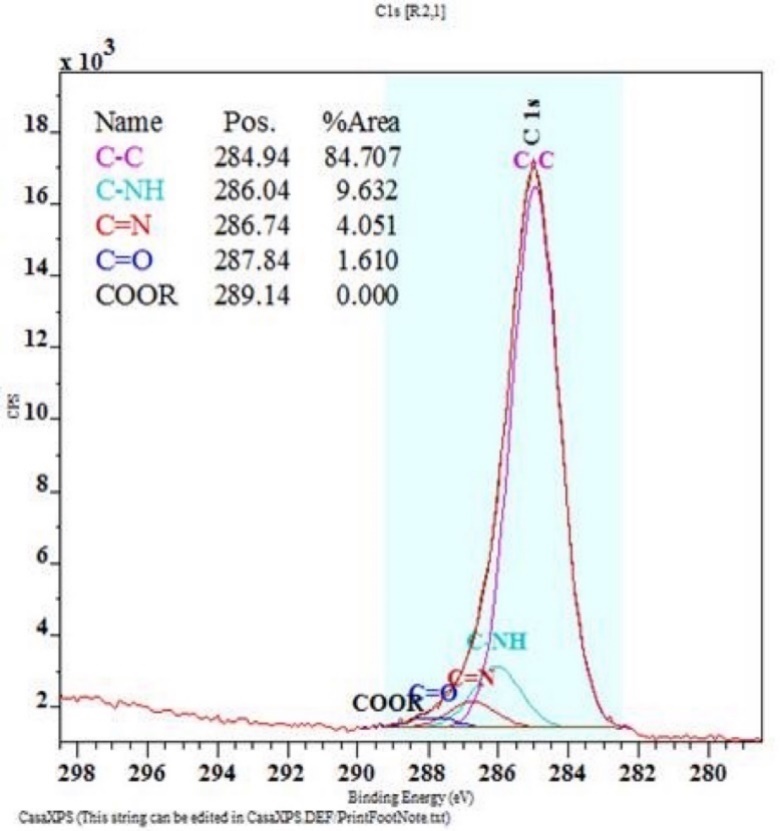

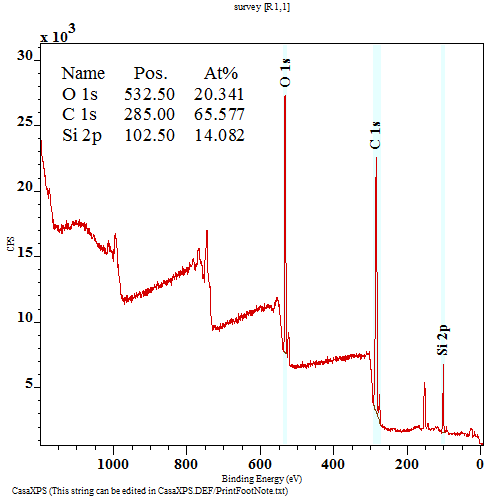

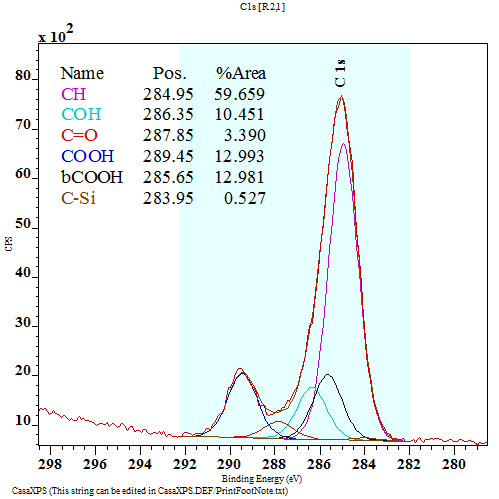


**a**

**b**

**d**

**c**
